# Supplementary material for: Saccharomyces boulardii (CNCM I-745) improves intestinal damage in sepsis by remodeling intestinal flora structure
Source: Front Cell Infect Microbiol. 2025 Jul 31;15:1602792. doi: 10.3389/fcimb.2025.1602792 (PMC12350329; doi:10.3389/fcimb.2025.1602792)
Supplement: Supplementary file 1 [file DataSheet1.zip › summary/summary/8_Advanced_analyse/2_PICRUSt2_result/METACYC/Group.DW4_vs_DW0/PICRUSt2_METACYC_Group.DW4_vs_DW0_diff.pdf]

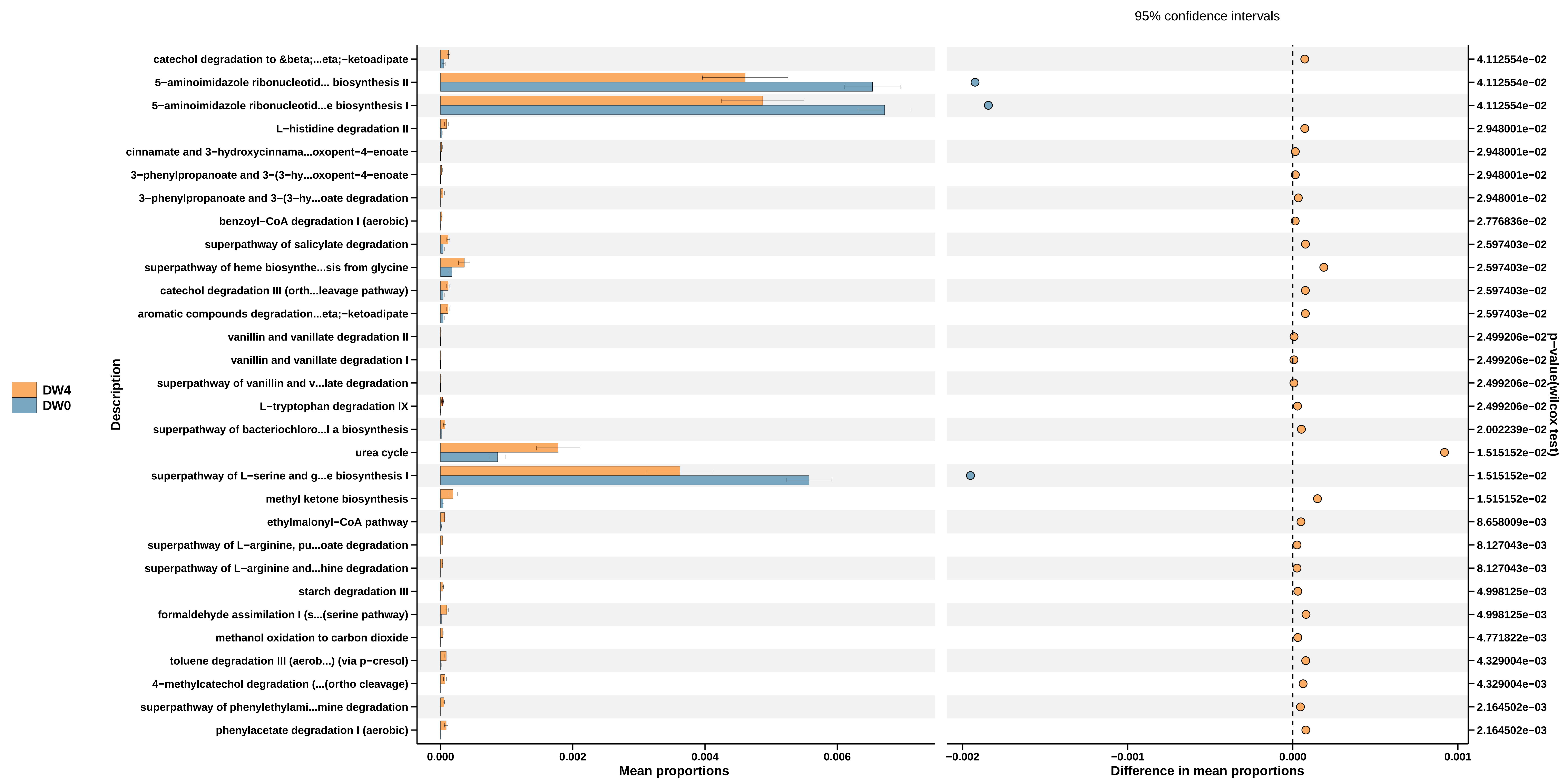
95% confidence intervals
DW4
DW0
Description
catechol degradation to β...eta;−ketoadipate
5−aminoimidazole ribonucleotid... biosynthesis II
5−aminoimidazole ribonucleotid...e biosynthesis I
L−histidine degradation II
cinnamate and 3−hydroxycinnama...oxopent−4−enoate
3−phenylpropanoate and 3−(3−hy...oxopent−4−enoate
3−phenylpropanoate and 3−(3−hy...oate degradation
benzoyl−CoA degradation I (aerobic)
superpathway of salicylate degradation
superpathway of heme biosynthe...sis from glycine
catechol degradation III (orth...leavage pathway)
aromatic compounds degradation...eta;−ketoadipate
vanillin and vanillate degradation II
vanillin and vanillate degradation I
superpathway of vanillin and v...late degradation
L−tryptophan degradation IX
superpathway of bacteriochloro...l a biosynthesis
urea cycle
superpathway of L−serine and g...e biosynthesis I
methyl ketone biosynthesis
ethylmalonyl−CoA pathway
superpathway of L−arginine, pu...oate degradation
superpathway of L−arginine and...hine degradation
starch degradation III
formaldehyde assimilation I (s...(serine pathway)
methanol oxidation to carbon dioxide
toluene degradation III (aerob...) (via p−cresol)
4−methylcatechol degradation (...(ortho cleavage)
superpathway of phenylethylami...mine degradation
phenylacetate degradation I (aerobic)
0.000
0.002
0.004
0.006
Mean proportions
−0.002
−0.001
0.000
0.001
Difference in mean proportions
p−value(wilcox test)
4.112554e−02
4.112554e−02
4.112554e−02
2.948001e−02
2.948001e−02
2.948001e−02
2.948001e−02
2.776836e−02
2.597403e−02
2.597403e−02
2.597403e−02
2.597403e−02
2.499206e−02
2.499206e−02
2.499206e−02
2.499206e−02
2.002239e−02
1.515152e−02
1.515152e−02
1.515152e−02
8.658009e−03
8.127043e−03
8.127043e−03
4.998125e−03
4.998125e−03
4.771822e−03
4.329004e−03
4.329004e−03
2.164502e−03
2.164502e−03
